# Feasibility of Hemolytic Disease of the Fetus and Newborn Case Ascertainment and Assessing Its Impact on Prenatal and Postnatal Outcomes: Protocol for Observational Studies

**DOI:** 10.2196/77836

**Published:** 2025-12-09

**Authors:** Nana A Mensah, Michael J Fassett, Nehaa Khadka, Jiaxiao M Shi, Fagen Xie, Vicki Y Chiu, Theresa M Im, Sunhea Kim, Daniella Park, Carol Mao, Matthew Molaei, Iris Lin, Darios Getahun

**Affiliations:** 1 Department of Research & Evaluation Kaiser Permanente Southern California Pasadena, CA United States; 2 Department of Obstetrics & Gynecology Kaiser Permanente West Los Angeles Medical Center Los Angeles, CA United States; 3 Department of Clinical Science Kaiser Permanente Bernard J. Tyson School of Medicine Pasadena, CA United States; 4 Johnson & Johnson Horsham, PA United States; 5 Department of Health Systems Science Kaiser Permanente Bernard J. Tyson School of Medicine Pasadena, CA United States

**Keywords:** hemolytic disease of the fetus and newborn, HDFN, hemolytic disease, fetus, newborn, protocol, epidemiology, treatment use, electronic health record

## Abstract

**Background:**

Hemolytic disease of the fetus and newborn (HDFN) is a rare but serious condition caused by maternal-fetal red blood cell antigen incompatibility. In an affected pregnancy, maternal immunoglobulin G antibodies cross the placenta and target fetal or neonatal red blood cells, leading to hemolysis, hyperbilirubinemia, and anemia. Although routine screening and alloimmunization prevention programs have contributed to the decline in HDFN in the United States, further understanding of its epidemiology is still needed.

**Objective:**

This protocol aims to provide an overview of the study design, methodology, and analytical approach used to investigate the epidemiology, treatment, and health care resource use of HDFN within a large integrated health care system.

**Methods:**

We conducted a retrospective cohort study of pregnant women who received obstetric care in the Kaiser Permanente Southern California (KPSC) health care system from January 1, 2008, to June 30, 2022. To identify HDFN cases, we used a novel methodology developed by KPSC researchers combining structured data and detailed clinical information extracted from unstructured records via a natural language processing–assisted chart review process. Chi-square and Wilcoxon rank sum tests were used to compare the distributions of maternal and infant demographic characteristics, as well as medical and perinatal conditions, by HDFN status. We also evaluated the association between HDFN and adverse perinatal outcomes using logistic regression models. Planned analyses using this unique cohort will include describing the annual prevalence, health care resource use, and treatment patterns of mothers and infants by HDFN status.

**Results:**

The study population consisted of 464,711 pregnancies, of which 136 (0.03%) were HDFN cases confirmed by chart review, resulting in 138 (0.03%) births (n=137, 0.99% live births and n=1, 0.01% stillbirth). The mean age at pregnancy was 29.8 (SD 5.7) years, and the population was racially and ethnically diverse.

**Conclusions:**

We present an overview of the methodology developed by KPSC clinicians and researchers on the epidemiology, treatment, and health care resource use of HDFN within a large and demographically diverse population of pregnant women. Our novel methodology, combining both structured and unstructured data and a natural language processing–assisted chart review process, ensures the successful identification of true cases to carry out pharmaco-epidemiological studies.

**International Registered Report Identifier (IRRID):**

DERR1-10.2196/77836

## Introduction

Hemolytic disease of the fetus and newborn (HDFN) is a rare disease caused by maternal-fetal red blood cell antigen incompatibility. HDFN occurs when maternal immunoglobulin G antibodies cross the placenta and destroy the fetal or neonatal red blood cells, leading to perinatal morbidity and mortality if left untreated [1-4]. Numerous alloantibodies are implicated in HDFN, including anti-D, anti-E, and anti-C, with anti-D being the most common and clinically significant cause [1,5]. The clinical presentation of HDFN varies widely, ranging from mild immune reactions to more serious forms of HDFN, such as severe anemia, congestive heart failure, or intrauterine death [1,5-7]. Other complications, including hyperbilirubinemia, cholestasis, and kernicterus [8-10], and long-term complications, such as neurodevelopmental impairments and cardiovascular disease, have been reported [1,8,11-13].

Due to inconsistencies in the definitions and criteria used to ascertain HDFN, there is wide variability in the reported annual prevalence of HDFN, with estimates ranging from 3 to 80 cases per 100,000 births [1,14]. This variability is influenced by differences in diagnostic practices, population characteristics, and the availability of prophylactic interventions such as Rh immunoglobulin [1]. Prior approaches to defining HDFN in electronic health record (EHR) data have often relied on either a single indicator, such as HDFN-specific diagnostic codes [15], or a combination of indicators, such as laboratory results suggestive of HDFN (eg, maternal alloantibody titers), fetal complications stemming from HDFN (eg, hyperbilirubinemia, neonatal anemia, and fetal hydrops), or maternal-fetal treatment for HDFN (eg, exchange transfusion and intrauterine transfusion) [16-24]. While using a combination of indicators improves the accuracy of HDFN case identification, prior studies have varied widely in the specific set of indicators selected, limiting the generalizability of their findings. In addition, several studies have taken a narrow approach by concentrating on antigen-specific alloimmunization [16,17], which may not capture the full spectrum of HDFN cases; therefore, the estimates provided in such studies do not accurately represent the broader epidemiology of the disease. Finally, studies describing other important components of the epidemiology of HDFN, including treatment plans and health care resource use, have not received enough attention in the literature. Considering these limitations, improved clarification of the epidemiology and perinatal complications of HDFN is warranted. Therefore, we plan to conduct retrospective cohort studies to address knowledge gaps in the epidemiology, treatment, and health care resource use of HDFN. Using a combination of structured and unstructured data collected at the point of care over the proposed study period, Kaiser Permanente Southern California (KPSC) developed a natural language processing (NLP)–assisted chart review process to accurately identify and characterize this rare condition [25]. This paper presents an overview of the study design and characteristics of the study population, as well as the methodology developed by KPSC researchers to define and establish an HDFN cohort. It also outlines our strategy to investigate the epidemiology, treatment, and health care resource use associated with HDFN in a large, diverse, and integrated health care system.

## Methods

### Study Design, Setting, and Data Sources

This retrospective cohort study was performed at KPSC, an integrated health care delivery system that serves more than 4.8 million members across 15 hospitals and more than 235 medical offices. KPSC membership is broadly representative of the socioeconomic, racial, and ethnic diversity of Southern California’s population [26-28], with long-term retention of its members, exceeding 95% at 2 years and 87% at 5 years, which supports the generalizability and longitudinal strength of research conducted within this population. Data for this study were extracted from the KPSC’s comprehensive EHR system, which contains detailed patient-level data, including diagnostic and procedural codes, pharmacy and laboratory records, and member demographics and behavioral information from patients receiving inpatient and outpatient care. In addition to structured data, KPSC’s EHR database contains unstructured data, which include free-text clinical notes, radiology, pathology, imaging reports, and clinician-patient communications.

The KPSC EHR databases comprise a comprehensive and longitudinal data infrastructure that includes health plan enrollment information, inpatient and outpatient clinical encounters, external claims, laboratory results, and pharmacy dispensing records. The inpatient database captures all inpatient hospitalization visits and records admission and discharge dates; *International Classification of Diseases*, ninth and tenth revisions; clinical modification diagnosis, procedure, and discharge codes; and *Current Procedural Terminology* codes. The outpatient database captures all primary care outpatient clinic visits, urgent care, and emergency room visits, with corresponding *International Classification of Diseases* and *Current Procedural Terminology* codes. The external claims database captures all outpatient (clinic, urgent care, and emergency room) and inpatient visits by KPSC enrollees to non-KPSC facilities where KPSC is financially responsible for the care of the patients. The pharmacy database captures medications dispensed and refilled to KPSC enrollees with a pharmacy benefit plan at KPSC-owned pharmacies. All databases are linked through a unique medical record number assigned to each enrollee for life, precluding multiple counts of the same health event for individuals across sources. To enhance case identification and characterization of rare conditions, additional relevant information was extracted from the unstructured data via NLP. This approach has been validated in prior KPSC studies and shown to substantially improve the accuracy of case identification compared to diagnosis codes alone [25].

### Study Population and Selection Criteria

Medical records of pregnant women and their children born between January 1, 2008, and June 30, 2022 (n=572,328), were included in this study. We excluded pregnancies without KPSC health plan membership (n=89,254, 15.59%), those ending in elective abortion (ie, abortion done not for medical reasons; n=921, 0.16%), and pregnancies with ABO alloimmunization of the newborn without the diagnosis of HDFN (n=17,442. 3.05%). After applying the exclusions, a total of 464,711 (81.19%) pregnancies remained for analysis.

### Study Outcome

We identified HDFN cases through a novel, systematic approach that began with the selection of HDFN-specific diagnostic and related codes. Next, we assembled a range of candidate HDFN indicators from structured EHR data, including positive indirect Coombs laboratory test results, abnormal maternal antibody titer results (anti-Kell ≥4; other antibodies ≥8), HDFN diagnosis codes, receipt of blood transfusion or intravenous immunoglobulin treatment, neonatal jaundice and phototherapy, and rho (D) immune globulin injection. To enhance case detection, we then developed an NLP approach, in conjunction with detailed medical record reviews, to detect these candidate indicators from clinical notes. Details of our HDFN identification process, including the NLP-assisted chart review process, have been published previously [25].

### Data Analyses

#### Completed Analyses

We aimed to describe the epidemiology of HDFN, including the demographics and clinical presentation of the disease among those with pregnancy outcomes over the study period (January 1, 2008, to June 30, 2022). The demographic and clinical presentation at the time of pregnancy were compared between HDFN cases and controls, as defined by our criteria. Descriptive statistics were calculated for the variables of interest, both overall and stratified by predefined categories. For continuous variables, the mean (SD) and median (IQRs) were computed. All missing data were treated as a separate category (dummy variable), and no imputation was performed. Categorical variables were compared using chi-square tests, while continuous variables were analyzed using the Wilcoxon rank sum test.

#### Planned Analyses

In future studies, we will report trends in the prevalence of HDFN-associated pregnancies over the study period, with 3-year rates standardized to the age, race, and ethnicity distribution of pregnant women from 2014 to 2016. This standardization will allow for meaningful comparisons across time by accounting for demographic shifts in the underlying population. We will also compare health care resource use patterns between pregnancies and infants affected by HDFN and those without the condition, focusing on key use metrics, such as maternal length of hospital and intensive care unit stays, neonatal intensive care unit stays, emergency room visits, and urgent care visits.

Furthermore, we will use logistic regression models to estimate both crude and adjusted odds ratios to evaluate the association between HDFN and a range of adverse maternal, fetal, and neonatal outcomes. These outcomes will include, but are not limited to, fetal death, preterm birth (defined as delivery before 37 weeks of gestation), low Apgar score (<7 at 5 min), and neonatal jaundice. To account for potential confounding, the models will be adjusted for a comprehensive set of covariates, including demographic characteristics (eg, age, race and ethnicity, and socioeconomic status), behavioral factors (eg, smoking status and substance use), clinical comorbidities (eg, diabetes and hypertension), obstetric history (eg, parity and prior cesarean delivery), and health care use patterns (eg, number of prenatal visits and hospitalizations during pregnancy) [29].

### Ethical Considerations

The Institutional Review Board of KPSC approved the study and granted an exemption from the requirement for patient informed consent (13503). The proposed study involves analyses of existing EHR data (ie, secondary data analysis); therefore, no compensation was provided to study patients. All data created for this project are fully anonymized to ensure patient confidentiality. The resulting research database is password protected and accessible only to authorized research staff responsible for data management and analysis.

## Results

### Demographic Data

The study population comprised 464,711 pregnancies, among which 136 (0.03%) were identified as HDFN pregnancies, resulting in 138 (0.03%) births (n=137, 0.99% live births and n=1, 0.01% stillbirth). This corresponds to a prevalence rate of 29.3 per 100,000 pregnancies (Table 1).

Pregnancies affected by HDFN were significantly more likely to involve women who were aged ≥35 years (*P*=.003), from non-Hispanic White racial-ethnic group (*P*=.01), multiparous (*P*<.001), multigravida (*P*<.001), and covered by Medicaid or private insurance (*P*=.04; Table 1).

**Table 1:** Distribution of maternal demographic, medical, and obstetric characteristics based on HDFN^a^ status.

Maternal characteristics		Total (N=464,711)	HDFN (n=136)	Non-HDFN (n=464,575)	*P* value^b^	
Age at index date (y), mean (SD)		29.8 (5.7)	31.8 (5.3)	29.8 (5.7)	<.001	
**Age at index date (y), n (%)**						.003
	<20	21,437 (4.6)	3 (2.2)	21,434 (4.6)		
	20-29	193,523 (41.6)	40 (29.4)	193,483 (41.6)		
	30-34	152,571 (32.8)	51 (37.5)	152,520 (32.8)		
	≥35	97,180 (20.9)	42 (30.9)	97,138 (20.9)		
**Race or ethnicity, n (%)**						.01
	Asian or Pacific Islander	62,045 (13.4)	17 (12.5)	62,028 (13.4)		
	Hispanic	213,525 (45.9)	57 (41.9)	213,468 (45.9)		
	Non-Hispanic Black	36,344 (7.8)	10 (7.4)	36,334 (7.8)		
	Non-Hispanic White	126,026 (27.1)	52 (38.2)	125,974 (27.1)		
	Missing	1159 (0.2)	0 (0)	1159 (0.2)		
	Other or multiracial	5794 (1.2)	0 (0)	5794 (1.2)		
	Unknown	20,977 (4.5)	0 (0)	20,977 (4.5)		
**Insurance type, n (%)**						.04
	Medicaid	44,583 (9.6)	22 (16.2)	44,561 (9.6)		
	Commercial	386,724 (83.2)	101 (74.3)	386,623 (83.2)		
	Private	27,385 (5.9)	11 (8.1)	27,374 (5.9)		
	Other or unknown	6019 (1.3)	2 (1.5)	6017 (1.3)		
**Parity, n (%)**						<.001
	Multiparous	264,887 (57)	120 (88.2)	264,767 (57)		
	Nulliparous	140,722 (30.3)	11 (8.1)	140,711 (30.3)		
	Unknown	59,102 (12.7)	5 (3.7)	59,097 (12.7)		
**Gravidity, n (%)**						<.001
	Multigravida	326,956 (70.4)	126 (92.6)	326,830 (70.4)		
	Nulligravida	136,049 (29.3)	10 (7.4)	136,039 (29.3)		
	Unknown	1706 (0.4)	0 (0)	1706 (0.4)		
Gestational weight gain (kg), mean (SD)		12.5 (7.2)	11.6 (7.1)	12.5 (7.2)	.19	
**Renal disease, n (%)**						<.001
	No	456,372 (98.2)	128 (94.1)	456,244 (98.2)		
	Yes	8339 (1.8)	8 (5.9)	8331 (1.8)		
**Chronic hypertension, n (%)**						.045
	No	455,405 (98)	130 (95.6)	455,275 (98)		
	Yes	9306 (2)	6 (4.4)	9300 (2)		

^a^HDFN: hemolytic disease of the fetus and newborn.

^b^*P* values were obtained using chi-square test for categorical variables and 2-tailed Student *t* test for continuous variables.

### Clinical Data

HDFN-affected pregnant women were more likely to have a higher prevalence of renal disease (*P*<.001) and chronic hypertension (*P*=.045; Table 1). Their offspring were more likely to be born preterm (<37 weeks of gestation; *P*<.001) and to have low birthweight (≤2499 g; *P*<.003), smaller head circumference (*P*=.01), and neonatal jaundice (*P*<.001) compared to offspring from a pregnancy that was not complicated by HDFN (Table 2).

**Table 2:** Distribution of infant characteristics based on HDFN^a^ status.

Infant characteristics			Total (N=446,499)		HDFN (N=138)		Non-HDFN (N=446,361)		*P* value^b^	
**Preterm birth, n (%)**										<.001
	No	402,035 (90)		97 (70.3)		401,938 (90)				
	Yes	42,280 (9.5)		40 (29)		42,240 (9.5)				
	Missing	2184 (0.5)		1 (0.7)		2183 (0.5)				
**Birthweight (g), n (%)**										<.001
	<1500	5982 (1.3)		4 (2.9)		5978 (1.3)				
	1500-2499	25,780 (5.8)		18 (13)		25,762 (5.8)				
	2500-3999	366,607 (82.1)		110 (79.7)		366,497 (82.1)				
	≥4000	38,773 (8.7)		4 (2.9)		38,769 (8.7)				
	Missing	9357 (2.1)		2 (1.4)		9355 (2.1)				
Head circumference (cm), mean (SD)			34.0 (2.7)		33.5 (2.3)		34.0 (2.7)		.01	
**Neonatal jaundice, n (%)**										<.001
	No	283,942 (63.6)		48 (34.8)		283,894 (63.6)				
	Yes	162,557 (36.4)		90 (65.2)		162,467 (36.4)				

^a^HDFN: hemolytic disease of the fetus and newborn.

^b^*P* values were obtained using chi-square test for categorical variables and Student *t* test for continuous variables.

### Laboratory Data

While specific laboratory parameters are not detailed in this summary, the study includes data extracted from the KPSC EHR system, which captures antibody types associated with HDFN. Among these, anti-D was the most frequent identified antibody (47/95, 50%), followed by anti-E (26/95, 27%), anti-C (16/95, 17%), and anti-K (7/95, 7%). Notably, 100% (7/7) of antibodies among non-Hispanic Black individuals were anti-D. Anti-D prevalence was also observed in non-Hispanic White individuals (22/39, 56%), Hispanic individuals (16/39, 41%), and Asian or Pacific Islander individuals (2/10, 20%). Anti-E was most common among Asian or Pacific Islander individuals (7/10, 70%), followed by non-Hispanic White individuals (10/39, 26%) and Hispanic individuals (9/39, 23%). Anti-C was found in 21% (8/39) of non-Hispanic White individuals and 18% (7/39) of Hispanic individuals, while anti-K was identified in 8% (3/39) of non-Hispanic White individuals and 10% (4/39) of Hispanic individuals. Additionally, serologic and hematologic markers relevant to the diagnosis and monitoring of HDFN, such as hemoglobin levels, hematocrit, reticulocyte counts, and total bilirubin, were extracted from laboratory records for further analysis.

### Study Timeline and Dissemination Plan

The study was funded in October 2022. Initial data extraction started in February 2023 and was completed in November 2023. Our final analysis, which focuses on trends in the prevalence of HDFN and patterns of health care resource use, including costs associated with pregnancies and infants affected by HDFN, is expected to be completed in early 2026. We plan to disseminate these findings through peer-reviewed, open-access journals; presentations at professional societal conferences; and meetings with key stakeholders by late 2026.

## Discussion

### Anticipated Findings

We presented an overview of the methodology used to establish a cohort of patients with HDFN, along with a description of the epidemiology, study design, characteristics of the study population, and health care resource use associated with HDFN. Our comprehensive approach successfully identified an HDFN prevalence of 29.3 per 100,000 among a cohort of more than 450,000 pregnancies. We found that pregnant women affected by HDFN tended to be older, non-Hispanic White, insured through Medicaid or private insurance, multiparous, and multigravida. Furthermore, these pregnancies were associated with a higher prevalence of renal disease. Finally, infants born from HDFN-associated pregnancies had a higher likelihood of preterm birth, low birthweight, small head circumference, and neonatal jaundice. Among HDFN cases, anti-D was the most frequently identified antibody, followed by anti-E, anti-C, and anti-K. Notable heterogeneity in the distribution of antibodies associated with HDFN was observed across racial and ethnic groups. This variation highlights potential disparities in immunologic profiles and may have implications for targeted screening and management strategies.

There is currently no standardized approach to reporting the incidence rates of HDFN, with recent studies showing considerable variation in prevalence estimates. A retrospective cohort study of US birth data between 1996 and 2010 reported a prevalence of 1695 cases per 100,000 births [7]. Although this estimate is substantially higher than our observed prevalence of 29 per 100,000 births, critical difference in case ascertainment methods may explain the discrepancy.

The majority of alloimmunized women in our cohort were multiparous and older than 30 years, which aligns with findings from previous studies [30]. It is important to note that this demographic trend may reflect an increased cumulative exposure to immunizing events, such as prior pregnancies or blood transfusions, as well as potential age-related changes in immune function that heighten susceptibility to alloimmunization [31]. These observations underscore the importance of early screening and targeted monitoring strategies in high-risk pregnancies, particularly among older and multiparous women.

In our study, the integration of structured data with unstructured data through an NLP algorithm was critical for accurately identifying HDFN cases. However, the rarity of the disease presented several challenges during the cohort development phase, including the need for manual validation and expert adjudication to ensure diagnostic accuracy.

As there are no straightforward diagnostic codes to identify HDFN cases, selecting the appropriate isoimmunization codes to accurately identify HDFN cases was a major challenge. To overcome this, we adopted an inclusive approach, using a broad range of isoimmunization diagnostic codes to minimize the risk of missing true cases. Furthermore, extracting laboratory data for antibody screening and indirect Coombs tests often required careful manual review of medical records, as this information was not always readily available in the test result fields. Further challenges arose during the medical review phase, where conclusively ascertaining often proved difficult for cases, even after reviewing both maternal and infant records. In some instances, we found that isoimmunization codes had been incorrectly assigned to infants based solely on maternal antibody test results. Moreover, several HDFN-related symptoms and treatments, such as jaundice, kernicterus, and hydrops fetalis, overlap with symptoms of other unrelated medical conditions. Therefore, a thorough review was critical to accurately determine true cases. Overall, we resolved most of these challenges through expert clinician review of ambiguous cases.

In future studies, we plan to compare health care resource use between pregnancies and infants affected by HDFN and those without, focusing on maternal hospital and intensive care unit stays, neonatal intensive care unit stays, and emergency and urgent care visits. We will also assess the association between maternal comorbidities and the development of alloimmunization, as well as examine the links between HDFN and adverse maternal and infant outcomes, including, but not limited to, stillbirth and preterm birth. Additionally, we plan to examine whether the prevalence of alloimmunization varies across racial and ethnic groups and explore the potential influence of sociocultural and genetic factors. As all pregnant women in our study are part of the same integrated health care system, disparities in access to care are unlikely to explain any observed racial or ethnic differences. This supports further investigation into other contributing factors, such as biological predispositions, cultural practices, and environmental exposures.

### Strengths and Limitations

A major strength of this study is the comprehensive approach to defining HDFN within the EHR, which combined structured and unstructured data with extensive chart reviews. This novel methodology enabled effective case ascertainment and facilitated both prevalence estimation and more complex epidemiological analyses. As noted in previous research, reliance on diagnostic codes alone, especially for rare and complex conditions, may provide inaccurate estimates [32]. In our analysis, fewer than 6% of true HDFN cases were appropriately coded as such, while more than 15% of confirmed HDFN cases lacked any HDFN-specific diagnostic codes. Even with our novel HDFN case ascertainment methodology, there are a few limitations to acknowledge. First, we used data from member patients of a large integrated health care system in Southern California, and although comparable to the overall residents of Southern California [26,28], our findings may not reflect national HDFN rates. Second, the accuracy of data hinges on the completeness of information recorded in the EHR system, which may introduce some degree of bias.

Overall, the database compiled for this study and the methodology described to ascertain HDFN cases provide the opportunity to conduct pharmacoepidemiological studies on rare conditions such as HDFN. Our study cohort represents a large, socioeconomically diverse population, and the results of this study will provide updated epidemiological data regarding the prevalence, temporal trends, and health care resource use associated with HDFN.

### Conclusions

This study established a robust methodology and comprehensive cohort for identifying HDFN cases, enabling future pharmacoepidemiological research on rare conditions. By leveraging a large, socioeconomically diverse population, the findings offer updated insights into HDFN prevalence, trends, and health care use. There is considerable heterogeneity in the characteristics of pregnancies associated with HDFN compared to those without HDFN, reflecting variations in maternal demographics, clinical profiles, and pregnancy outcomes, underscoring the clinical significance and health care burden of HDFN. The approach described may serve as a foundation for generating evidence to inform clinical guidelines and support future drug development, including clinical trials.

## Abbreviations

EHR: electronic health record
HDFN: hemolytic disease of the fetus and newborn
KPSC: Kaiser Permanente Southern California
NLP: natural language processing
